# Leptin Promotes Angiogenesis via Pericyte STAT3 Pathway upon Intracerebral Hemorrhage

**DOI:** 10.3390/cells11172755

**Published:** 2022-09-03

**Authors:** Qi Cui, Yingmei Zhang, Ning Tian, Jiaxin Yang, Dongshan Ya, Wenjing Xiang, Zixian Zhou, Yanlin Jiang, Jungang Deng, Bin Yang, Xiaohui Lin, Qinghua Li, Rujia Liao

**Affiliations:** 1 Laboratory of Neuroscience, Affiliated Hospital of Guilin Medical University, Guilin Medical University, Guilin 541004, China; 2 Department of Neurology, Affiliated Hospital of Guilin Medical University, Guilin Medical University, Guilin 541004, China; 3 Guangxi Clinical Research Center for Neurological Diseases, Affiliated Hospital of Guilin Medical University, Guilin Medical University, Guilin 541004, China; 4 Department of Pharmacology, Affiliated Hospital of Guilin Medical University, Guilin Medical University, Guilin 541004, China; 5 Department of Geriatrics, Affiliated Hospital of Guilin Medical University, Guilin Medical University, Guilin 541004, China

**Keywords:** leptin, intracerebral hemorrhage, angiogenesis, pericyte, neuroprotection

## Abstract

Angiogenesis is a vital endogenous brain self-repair processes for neurological recovery after intracerebral hemorrhage (ICH). Increasing evidence suggests that leptin potentiates angiogenesis and plays a beneficial role in stroke. However, the proangiogenic effect of leptin on ICH has not been adequately explored. Moreover, leptin triggers post-ICH angiogenesis through pericyte, an important component of forming new blood vessels, which remains unclear. Here, we reported that exogenous leptin infusion dose-dependent promoted vascular endothelial cells survival and proliferation at chronic stage of ICH mice. Additionally, leptin robustly ameliorated pericytes loss, enhanced pericytes proliferation and migration in ICH mice in vivo, and in ICH human brain microvascular pericytes (HBVPC) in vitro. Notably, we showed that pericytes-derived pro-angiogenic factors were responsible for enhancing the survival, proliferation and tube formation followed leptin treatment in human brain microvascular endothelial cells (HCMEC/D3)/HBVPC co-culture models. Importantly, considerable improvements in neurobehavioral function and hostile microenvironment were observed in leptin treatment ICH mice, indicating that better vascular functionality post ICH improves outcome. Mechanistically, this study unveiled that leptin boost post-ICH angiogenesis potentially through modulation of leptin receptor (leptinR)/Signal Transducer and Activator of Transcription 3 (STAT3) signaling pathway in pericyte. Thus, leptin may be a lucrative option for the treatment of ICH.

## 1. Introduction

Intracerebral hemorrhage (ICH) is a devastating stroke subtype around world, which causes long-term and severe neurologic impairment for survivors despite multiple treatment approaches [1,2,3]. A growing number of evidence has shown that for angiogenesis, a vital endogenous brain self-repair processes, enhancing its processes may display favorable corrective impacts and possibilities in sparing the damaged brain tissue and advancing functional recovery after ICH [4,5,6,7]. The new blood vessels could be detected around the hemorrhagic region by stimulating these new vessels growth with lactate [8], nicotine [9], and natural agents [10,11,12], which could help stabilize brain perfusion, provide oxygen, glucose, and nutrients supplement to damaged brain regions, thereby promoting neuronal survival, brain plasticity, and neurologic recovery.

Leptin (LEP) is an adipocyte-derived endocrine hormone which plays a key part within the vitality homeostasis [13].

In expansion, it is included in direction of other physiological forms such as reproduction [14], immune function [15], and angiogenesis [16,17,18]. This idea that leptin is postulated to produce beneficial influences on angiogenesis was confirmed in most investigations, although a few opposed views are also discussed [19,20]. For example, ob/ob mice (leptin deficiency) lacking resistance to metastasis probably involves reduced angiogenesis [21]. Additionally, wound healing disorder (due to deficient angiogenesis) in ob/ob mice is corrected by both topical and systemic leptin administration [22]. In addition, leptin was found to directly induce endothelial cell proliferation and expression of matrix metalloproteinases activation of leptin receptor in vivo and in vitro [23]. Leptin can also potentiate vascular endothelial growth factor (VEGF) or Fibroblast growth factor 2 (FGF2) mediated angiogenesis [24]. Therefore, it seems that leptin as a potent angiogenic factor can play a beneficial role against ICH outcome. Noteworthily, angiogenesis includes coordinated events that involve vascular endothelial cells (ECs) and pericytes [25]. Pericytes is essential for forming new blood vessels. Pericytes’ recruitment by nascent vessels provide direct, basement-membrane penetrating contacts with ECs to providing stabilization and maturation signals [26,27]. Additionally, pericytes contribute to vessel maturation through the release of paracrine-acting growth factors [28]. Moreover, a complex web of bidirectional signaling pathways mediate angiogenesis actions for pericytes. For example, pericyte-derived angiopoietin-1 (Ang-1) is authoritative to Tie-2 tyrosine kinase receptors primarily on endothelial cells, advancing vessel formation by expanding endothelial cell expansion, movement, and survival [29]. Additionally, pericytes-derived platelet derived growth factor receptor beta (PDGFRβ) binding to platelet-derived growth factor BB on endothelial cells enhancing pericytes proliferation and recruiting pericytes to the endothelial tube [30]. Pericytes (paracrine signaling) secrete vascular endothelial growth factor A (VEGFA) that activates the vascular endothelial growth factor receptor 2 (VEGFR2) pathway to promotes angiogenesis [31]. It is vital to note that past considerations appear that leptin’s proangiogenic impacts dependent activation of VEGFR2 receptor or synergistically with the key angiogenic mediators like fibroblast growth factor-2, VEGF, and its receptor VEGFRα, invigorating vascular porousness, consequently coming about in useful angiogenesis [32]. Importantly, brain pericytes express leptin receptors [33], and secrete abundant levels of LEP to exert autocrine promotion of pericyte survival and migration as well as paracrine stimulation of ECs proliferation, permeability, and network formation [34,35]. Nevertheless, the angiogenesis functional relevance of the LEP pathway in pericytes under ICH condition remains unknown.

Given the effect of leptin’s angiogenesis, and the angiogenesis useful pertinence of the LEP pathway in pericytes beneath ICH condition remains obscure, we hypothesized that leptin includes a neuroprotection impact on chronic phase of ICH in terms of post-ICH angiogenesis by means of activating LEP pathway in pericyte. Herein, we show that leptin directly impacts angiogenesis and outcome at a later stage of ICH. Leptin ameliorates pericytes loss and enhances pericytes proliferation and migration following ICH in vivo and in vitro. Importantly, activation of pericytes STAT3 signaling pathway and induction of pericytes-derived angiogenic factors is responsible for leptin proangiogenic after ICH. These findings could lead to a better understanding of ICH-related events and a novel post-ICH therapy strategy.

## 2. Materials and Methods

### 2.1. Animals

All animal tests and procedures were approved by the Guilin Medical University Animal Experimentation Committee (permission number GLMC202003006) and followed the National Institutes of Health Guide for the Care and Use of Laboratory Animals. In this investigation, adult male mice from the C57BL/6j strain were employed. All animals were acquired from the Hunan SJA laboratory Animal Co., Ltd. (Changsha, China).

### 2.2. Animal Groups

Animals were randomly divided into five groups: (1) SHAM group, (2) ICH group, (3) three leptin treatments groups. Each group was further randomly divided into their subgroups, named as 1d, 7d, and 14d subgroups. Animals were administered leptin at dose of 1, 10, 20 μg per mice once a day for 7 days (Lateral ventricle injection, icv; MAB498-100, R&D Systems, Emeryville, CA, USA). The dosage of drugs was selected based on previous studies with minor change [36]. Leptin was administered immediately after the induction of ICH. The Sham group was administered the same volume of 0.9% saline. The animals were sacrificed, and brain tissue was obtained at each terminal time point.

### 2.3. ICH Model

Intracerebral hemorrhage was established by injection of autologous blood into the caudate nucleus according to our previously report [37,38]. Briefly, mice were anesthetized with sevoflurane and fixed on a mouse stereotaxic brain locator. A total volume of 30 μL autologous blood from the tail artery without anticoagulant was injected into the right basal ganglia (coordinates: 0.1 mm anterior, 1.0 mm posterior, and 3.0 mm depth) at a delivery rate of 2 μL/min using a micro-infusion pump (RWD Life Science, Shenzhen, China). The infusion needle was kept in place for an extra 10 min after infusion, and the wound was closed. For the SHAM group, a total volume of 30 μL of 0.9% saline was infused instead of tail artery blood.

For ICH in vitro model, hemin-induced cell death was used as previously reported [37]. Briefly, cells were seeded at density in vessel for time and then exposed to 100 μM hemin (BCBR5047V, Sigma Aldrich Co., St. Louis, MO, USA) for 24 h to induce cell death.

### 2.4. Neurological Deficit Score

The neurological function evaluation of the mice was performed using the Modified Garcia test before being killed at each corresponding time point. The evaluation consists of the following six tests: Spontaneous Activity (0–3), Symmetry in the Movement of Four Limbs (0–3), Forepaw Outstretching (0–3), Climbing (0–3), Body Proprioception (0–3), and Response to Vibrissae Touch (0–3). The lower the score, the more severe the neurological deficit.

### 2.5. Rotarod Test

For the rotarod test, according to our previously report [39], mice were placed on an accelerating rotarod cylinder (Xinruan Instruments, Shanghai, China), which gradually accelerated from 0 to 40 rpm within 1.5 min, and the test lasted for five minutes. Latency to falling off during the procedure was recorded.

### 2.6. Grid Walking Test

To evaluate deficiencies in mouse descending motor control, the grid walking test is employed. Animal is placed on top of a floor grid that has 1 cm-wide holes. The grid is elevated on a clear plexiglass platform and surrounded by clear plexiglass walls. In order for a camera view from above to see the grid floor as a line seen from both sides, mirrors perpendicular to each other are positioned at an angle on two of the sides of the apparatus. For a predetermined period of time, the mouse is free to explore the container, and the behavior is noted. The number of foot slides through the grid can then be determined by analyzing the footage. The two angles of the mirror allow the experimenter to assess which foot had slipped.

### 2.7. Open Field Test

Animals were exposed to a white plywood 50 by 50 cm circular arena with nine parts on the floor. Mice were individually placed in the apparatus’s corner, and a digital camera was positioned above their head to record their behavior for five minutes. Tests were carried out seven and fourteen days after operation. The average speed was analyzed using GraphPad Prism 8.0.2 software package.

### 2.8. Pericyte and Vascular Endothelial Cell Culture

Human brain microvascular pericytes (HBVPC, ZQ0993), human brain microvascular endothelial cells (HCMEC/D3, ZQ0961), brain microvascular pericyte culture medium (ZQ-1321), and brain microvascular endothelial cell culture medium (ZQ1304) were purchased from Shanghai Zhong Qiao Xin Zhou Biotechnology. The culture medium consisted of basal culture medium, 5% serum, and growth factors of respective cells. Cells were adherent cultured at 37 °C in a 5% CO_2_ incubator. When the cells reached about 90% degree of fusion, they were digested with 0.25% trypsin and passed for culture.

### 2.9. Pericyte and Vascular Endothelial Cell Co-Culture System

Pericytes were inoculated in the Transwell chamber of the 6-well plate, and vascular endothelial cells were inoculated in the 6-well plate. The non-contact co-culture model was established in a 0.4 μm Transwell chamber and placed at 37 °C with a volume fraction of 5% CO_2_ culture in incubator.

### 2.10. Cell Viability Assay

Pericytes or co-cultured endothelial cells were inoculated into 96-well plates and the cell density was adjusted to 4 × 10^4^ cells/mL. Cells were treated DMEM (Gibco, Shanghai, China), Hemin, or various concentrations of leptin (0.1/1/10 ng/mL). Following incubation for 24 h, 10 μL of CCK-8 (MCE, HY-K-0301, Monmouth Junction, NJ, US) reagent was added to every well and then cultured for 2 h. In all cases, each experiment was performed in triplicate. The absorbance was analyzed at 450 nm using a Synergy™ HT Multi-Mode Microplate Reader (Biotek, Winooski, VT, USA).

### 2.11. Scratch Test

Firstly, a straight and uniform horizontal line was drawn behind the 6-well plate with a marker, with at least 5 lines in each well. Approximately 5 × 10^5^ cells were added to each well and cultured for 24 h. Use the tip of the gun to compare the ruler, and scratch perpendicular to the horizontal line behind: wash the cells with PBS for 3 times, remove the scratched cells, add serum-free medium to take photos and treat with various drugs. After incubation in a cell incubator for 24 h, the ImageJ v2021.8.0 software (National Institutes of Health, Bethesda, MD, USA) was used for analysis after photos were taken.

### 2.12. Tube Formation Assay

Tube formation assay was performed to assess the angiogenic impact of leptin. All test materials and pipettes were prechilled, and after that a 96-well plate was coated with 50 μL of growth factor-reduced Matrigel (Corning, 082704, Shanghai, China). The plates were placed in the incubator for 30 min until Matrigel was fully solidified. Treated HCMEC/D3 were collected, and 1 × 10^4^ cells were inoculated in each well, with 3 multiple wells in each group. The 96-well plate was placed in an incubator at 37 °C with 5% CO_2_ for 6 h, observed under a microscope, and photographed for each hole, selected for 5 fields of vision. The number of bifurcation points of tubular structures was recorded. Bifurcation points represented the degree of tubular formation in vitro, and the average value was determined. Measure the capacity of each component tube.

### 2.13. 5-Ethynyl-2-Deoxy Uridine (EdU) Incorporation Assay

Cells were collected at logarithmic stage, cultured in 24-well plates at a density of 1 × 10^4^ cells per well along with EdU (Cell-light™ Apollo 567 Stain Kit, Guangzhou RiboBio Co., Ltd., Guangzhou, China) at a working concentration of 50 μm in 100 μL culture medium for 2 h. The cells were washed twice in phosphate-buffered saline (PBS) for 5 min on each event, at that point fixed with 4% formaldehyde for 30 min at room temperature, permeabilized by incubation with 0.5 % Triton X-100 (Sigma, Ronkonkoma, NY, USA,500 μL per well) in PBS for 10 min, then the next steps were continued as per the instructions. After the staining, the cells were imaged beneath a fluorescent microscope (Leica Dm2500, Leica Co., Heidelberg, Germany). All the assays were repeated using 6 cell samples.

For tissue samples, EdU (5 mg/kg, in saline, Guangzhou RiboBio Co., Ltd.) was injected twice daily for seven days after ICH. Mice were perfused with 0.9% saline, followed by 4% paraformaldehyde on the seventh day after ICH. The brains were removed and then subjected to cut 10μm frozen slices. The following steps are the same as the steps in cells.

### 2.14. SYTOX Green Fluorescence Staining

Cell death was determined with a SYTOX-green fluorescent probe (S11348, Thermo Scientifc, Waltham, MA, USA). Briefly, all treated cells were incubated with 5 μM SYTOX-green fluorescent probe for 5 min at room temperature. SYTOX-green fluorescent and bright field pictures were taken beneath the fluorescence microscope, and an examiner blinded to treatment group calculated the number of dead cells with ImageJ v2021.8.0 (National Institutes of Health, Bethesda, MD, USA).

### 2.15. Histopathology

For brain tissues, mice were transcardially perfused with 0.9% saline at 4 °C, taken after 4% paraformaldehyde. Brains were removed and postfixed in 4% paraformaldehyde overnight, and after that immersed in 30% sucrose for 48 h at 4 °C. The tests were embedded in OTC glue, cut into 8 μm pieces using a cryostat (CM 3050S, Leica, Wetzlar, Germany), and mounted on coated glass slides.

Hematoxylin and eosin (H&E) were utilized to recolor the pieces. Histopathological changes of the tests were observed, taken after a blind procedure.

To evaluate brain neurons wounds initiated through ICH, Nissl staining was performed following the manufacturer’s protocol (Beyotime Institute of Biotechnology, Shanghai, China).

### 2.16. Immunofluorescence 

Brain tissues samplers and cells were washed 3 times with PBS and permeabilized with Triton X-100 (0.1%). Next, samples were incubated with anti-PDGFRβ (1:200; RP7212, GeneTex, CA, USA), anti-NG2 (1:200; DF12589, Affinity, Changzhou, China), anti-p-STAT3 (1:200, AF3295, Affinity, Changzhou, China), and anti-NeuN (1:200; DF6145, Affinity, Changzhou, China) at 4 °C overnight. After washing with PBS, the tests were hatched with Alexa Fluor-conjugated secondary antibodies (anti-rabbit 488 or anti-rabbit 594, 1:200, Affinity, Changzhou, China) for 1 h at room temperature, followed by washing with PBS and then incubation with DAPI for 2 min. Images of stained sections were observed with a fluorescence microscope (Leica DM2500, Leica Co., Heidelberg, Germany) and analyzed using ImageJ software.

### 2.17. Western Blotting

Brain protein samples were collected for detecting leptinR, P-STAT3, STAT3, VEGF, VEGFA, VEGFR2, NG2, p-JAK1, JAK1, Cyclin D2, CDK2, Rac1, RhoA, Cdc42, and 4-HNE level. Brain samples were isolated and homogenized in an ice-cold RIPA lysis buffer, according to the manufacturer’s instruction. Nuclear extraction was separated using a nuclear and cytoplasmic protein extraction kit (Beyotime, Shanghai, China). Equal amounts of protein per sample were loaded and separated by SDS–PAGE and blocked with 5% skimmed milk for 1 h in room temperature. Then, the membranes were probed with primary antibodies against leptinR (1:1000, DF7139, Affinity, Changzhou, China), P-STAT3 (1:1000, AF3295, Affinity, Changzhou, China), STAT3 (1:1000, AF6294, Affinity, Changzhou, China), VEGFA (1:1000, AF5131, Affinity, Changzhou, China), VEGFR2 (1:1000, AF6281, Affinity, Changzhou, China), NG2 (1:1000, DF12589, Affinity, Changzhou, China), and 4-HNE (1:2000, AB46545, Abcam, Cambridge, UK),VEGF (1:500, GTX48811, GeneTex)p-JAK1 (1:1000, AF2012, Affinity, Changzhou, China), JAK1 (1:1000, AF5012, Affinity, Changzhou, China), Cyclin D2 (1:1000, AF5410, Affinity, Changzhou, China), CDK2 (1:1000, AF6237, Affinity, Changzhou, China), Rac1 (1:1000, AF4200, Affinity, Changzhou, China), RhoA (1:2000, AB86297, Abcam, Cambridge, UK), Cdc42 (1:2000, AB155940, Abcam, Cambridge, UK) at 4 °C overnight. The membranes were washed with PBS with Tween 3 times, followed by incubation with horseradish peroxidase-conjugated anti-mouse (1:2000, CST, Beverly, MA, USA) or anti-rabbit antibody (1:2000, CST) for 1 h at room temperature. An enhanced chemiluminescence detection system (Beyotime Biotech, Shanghai, China) was used to detect the bands. Specific binding was tested and detected by a Gel Imaging System (Azure c300) and normalized to GAPDH using Image J software.

### 2.18. Real-Time PCR

Quantitative real-time RT-PCR evaluation for the messenger RNA (mRNA) levels was conducted through utilizing Prime Script RT-PCR kits (RR047A and RR820A, Takara) according to the manufacturer’s instructions. The mRNA level of GAPDH was used as an internal control. The real-time PCR program steps: Stage 1: Activation: 50 °C for 2 min; Stage 2: pre-soak: 95 °C for 10 min; Stage 3: Denaturation: 95 °C for 15 s, annealing: 60 °C for 1 min; Stage 4: Melting curve: 95 °C for 15 s, 60 °C for 15 s, 95 °C for 15 s. The mRNA level of each target gene was normalized to that of GAPDH mRNA. Fold-induction was calculated using the 2^−ΔΔCT^ method, as previously described [40]. The specific sequences of primers used were shown as follows: for mouse DLL4, Forward primer 5′-TTCCAGGCAACCTTCTCCGA-3′; Reverse primer 5′-ACTGCCGCTATTCTTGTCCC-3′; for mouse MYC, Forward primer 5′-ATGCCCCTCAACGTGAACTTC-3′; Reverse primer 5′-CGCAACATAGGATGGAGAGCA-3′; for mouse ESM1, Forward primer 5′-CTGGAGCGCCAAATATGCG-3′; Reverse primer 5′-TGAGACTGTACGGTAGCAGGT-3′; for mouse Rac1, Forward primer 5′-GAGACGGAGCTGTTGGTAAAA-3′; Reverse primer 5′-ATAGGCCCAGATTCACTGGTT-3′; For mouse GAPDH, Forward primer 5′- AGGTCGGTGTGAACGGATTTG-3′ACTGTACGGTAGCAGGT3′. Reverse primer 5′-TGTAGACCATGTAGTTGAGGTCA-3′.

### 2.19. GSH and SOD Measurement

The ELISA detection kits were selected for total superoxide dismutase (SOD, BC0175, Solarbio, Beijing, China), total glutathione peroxidase (GSH, BC1175, Solarbio, Beijing, China) measurement, respectively. According to the manufacturer’s instructions, brain tissues were disconnected and placed into an ice-cold 0.9% normal saline. Then, the samples were homogenized, followed by centrifugation at 3000 rpm for 15 min. Supernatant was collected for GSH and SOD measurement using a Synergy™ HT Multi-Mode Microplate Reader (Agilent Biotek, Santa Clara, CA, USA).

### 2.20. Statistical Analysis

All results are displayed as mean ± SEM. Significant differences were calculated by one-way ANOVA, with Bonferroni correction for post hoc comparisons between multiple experimental groups. Student’s t test was applied for other comparisons between two groups. *p* < 0.05 was considered to be statistically significant. “*n*” stands for the number of mice used in the experiment. Statistical analysis was performed using GraphPad Prism 8.0.2 software package.

## 3. Results

### 3.1. Leptin Dose-Dependent Drives Remarkable Angiogenesis and Alleviates Neurological Dysfunction after ICH 

**Figure 1:**
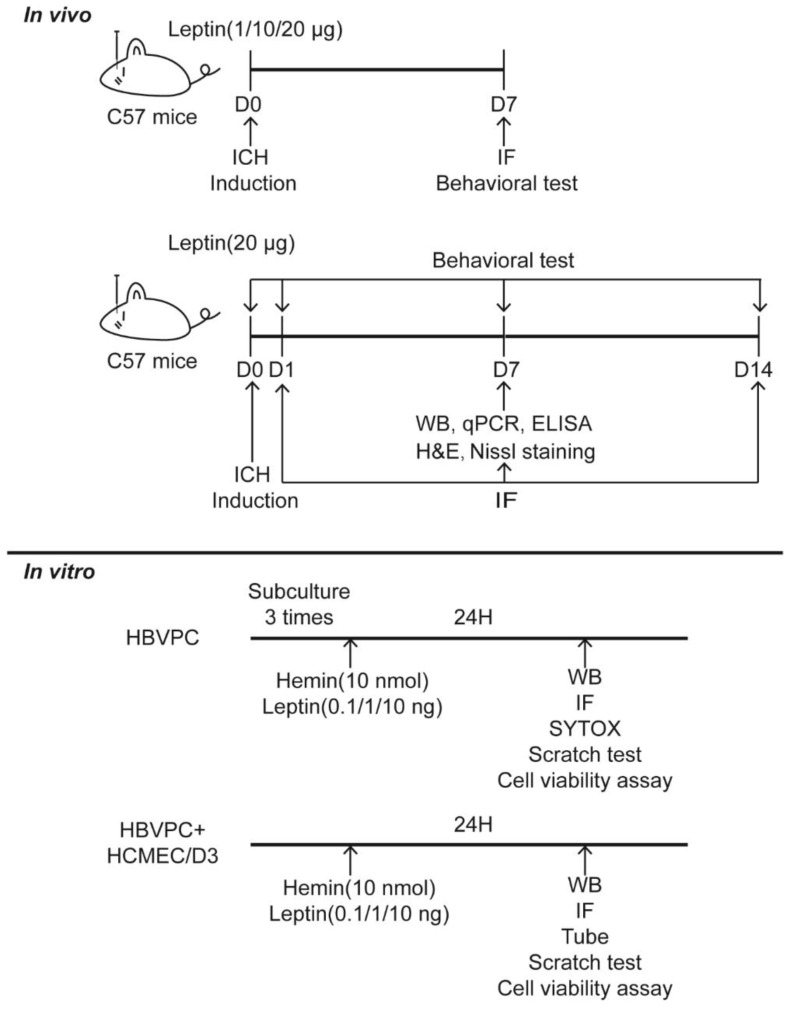
The experimental design in this study. D: day.

**Figure 2:**
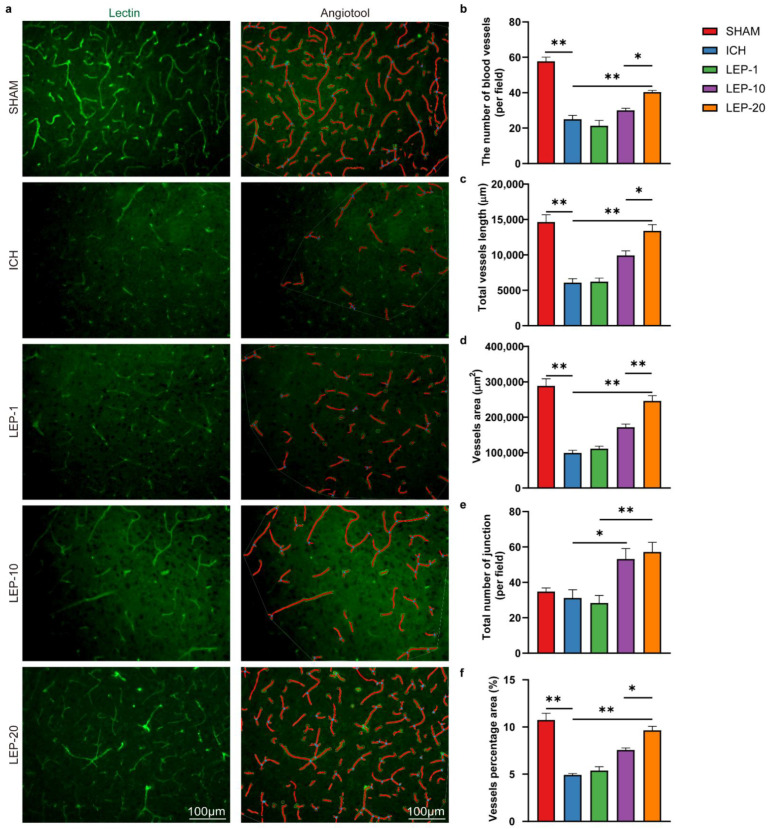
Leptin drives neoangiogenic in a dose–dependent manner. (**a**) Representative immunofluorescence staining images of vascular endothelial cells marker lectin (green) at day 7 after ICH. AngioTool software was used to make quantitative measurements and perform analyses as illustrated (in red). The number of blood vessels (**b**), total vessels length (**c**), vessels area (**d**), total number of junction (**e**), and vessels percentage area (**f**) were quantified. *n* = 6 per group. * *p* < 0.05, ** *p* < 0.01, between the indicated groups. Error bars, mean ± SEM.

The pathophysiological occasions taking after ICH are complicated, i.e., the acute excitotoxicity and inflammatory infiltration ordinarily take place inside 3 days after the onset of hemorrhage, while the angiogenesis and neurogenesis regularly show up after that. Previous studies found that angiogenesis was vigorous from days 7 to 14 in the rodent brain after ICH [8]. Thus, at first, we experimented the doses of leptin (1, 10, or 20 μg), which are often selected dosages in the study of stroke, and chose day 7 to evaluate the pro-angiogenesis properties of leptin. Additionally, to identify the effect of leptin on angiogenesis after ICH, the fixed brain tissues vascular were stained with lectin, and the number of blood vessels, total vessels length, vessels area, total number of junctions, and vessels percentage area were analyzed with AngioTool 0.6 software (National Institutes of Health, Bethesda, MD, USA) according previous reports [41]. The experimental design is shown in Figure 1. We found that the highest dose provided the most prominent pro-angiogenic effect as evidenced by the number of blood vessels, total vessels length, vessels area, total number of functions, and vessels percentage area (Figure 2).

**Figure 3:**
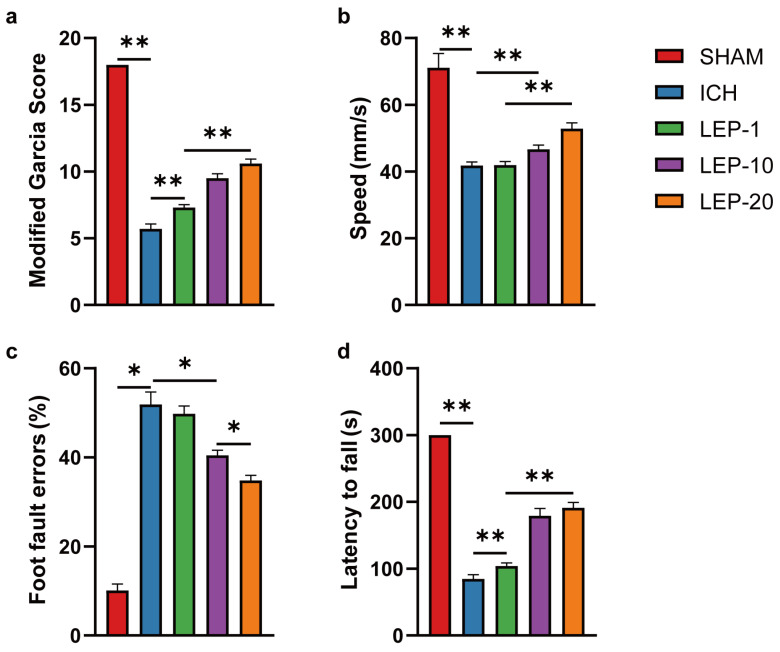
Leptin dose-dependent improved hemorrhagic-induced brain neuro-dysfunction. (**a**) The assessment of the modified Garcia score was performed at 7 days after ICH. The open field test (**b**), the foot faults of left forelimb in the grid walking test (**c**), and the rotarod test (**d**) were performed at 7 days after ICH. *n* = 10 per group. * *p* < 0.05, ** *p* < 0.01, between the indicated groups. Error bars, mean ± SEM.

Under different treatment regimens, we next evaluated the neuroprotection effects of leptin on neurological performance. Neurological deficit scores were detected at 7 days after ICH. The results showed that neurological score was significantly reduced in ICH mice. After receiving lateral ventricle leptin injections, the scores were increased in a dose-dependent manner at 7 days after ICH (Figure 3a). In the open field test, we found that the speed in ICH mice was markedly decreased. However, the speed was significantly increased with dose-dependent leptin treatment compared with that of the saline group (Figure 3b). In the grid walking test, the ICH mice showed higher in the ratio of foot fault at 7 days. As expected, dose-dependent leptin treatment decreased the ratio induced by ICH (Figure 3c). In the rotarod test, the mice appeared severed motor function disability compared to the sham group, as indicated by a diminish in the latency to fall. Leptin treatment dose-dependently essentially prevented the diminish at 7 days after ICH (Figure 3d).

Altogether, these data indicate that dose-dependent leptin treatment could promote angiogenesis and ease behavioral impairment in mice 7 days after ICH injury. In addition, leptin 20 μg treatment displayed the most robust pro-angiogenic effect and protection on neurological function. Thus, 20 μg was chosen as the dosage for the subsequent experiments.

### 3.2. Leptin Promotes Angiogenesis and Neurological Recovery at Later Stage of ICH

**Figure 4:**
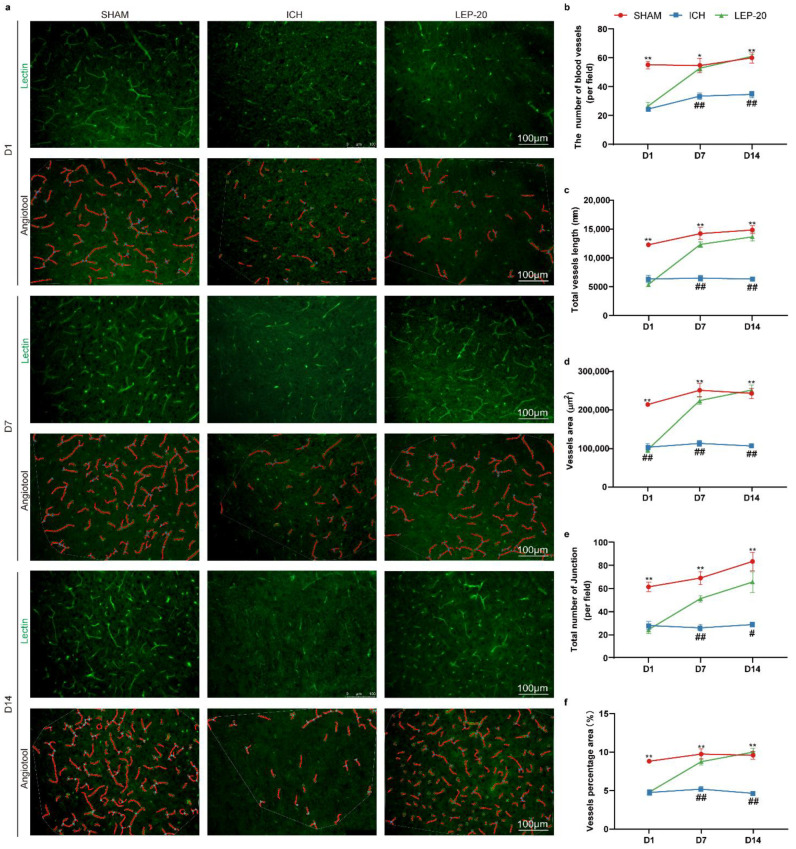
Leptin treatment drives neoangiogenic at different stages of ICH. (**a**) Representative immunofluorescence staining images of vascular endothelial cells marker lectin (green) at 1, 7, and 14 days after ICH. AngioTool software was used to make quantitative measurements and perform analyses as illustrated (in red). The number of blood vessels (**b**), total vessels length (**c**), vessels area (**d**), total number of junction (**e**), and vessels percentage area (**f**) were quantified in indicated days, respectively. *n* = 6 per group. * *p* < 0.05, ** *p* < 0.01 versus Sham group; # *p* < 0.05, ## *p* < 0.01 versus ICH group. Error bars, mean ± SEM. D: day.

Previous studies have indicated that leptin aggravates brain injury at the acute phase of ICH via promoting inflammatory response [26]. However, the role of leptin in the chronic stage of ICH remains unclear. In parallel, our present study has showed a great neuroprotective effect of leptin after ICH. Thus, we further detected the pro-angiogenic effect as well as neurological behavior in different stages of ICH. The angiogenesis was then calculated at 1, 7 and 14 days after ICH in lectin staining images. The results of angiogenesis quantitative analysis revealed that leptin treatment mice exhibited a statistically significantly increased number of blood vessels, total vessels length, vessels area, total number of junctions, and vessels percentage area than control mice at day 7 and day 14 following ICH. In contrast, leptin treatment has no effect on angiogenesis at 1 day after ICH (Figure 4).

**Figure 5:**
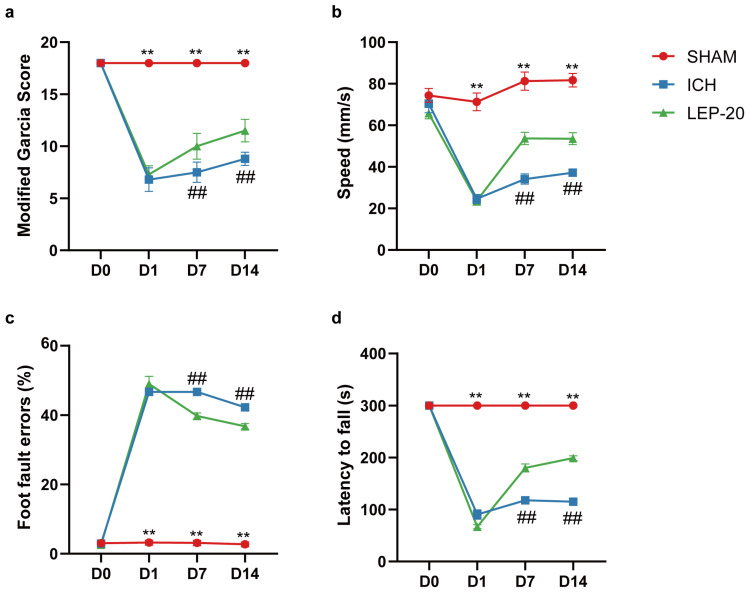
Leptin treatment improved brain neuro-dysfunction at different stages of ICH. (**a**) Modified Garcia score was performed at pre-operation and post-ICH days 1, 7, and 14 after ICH. The open field test (**b**), the foot faults of left forelimb in the grid walking test (**c**), and the rotarod test (**d**) were performed at pre-operation and post-ICH days 1, 7, and 14 days after ICH. *n* = 10 per group. ** *p* < 0.01 versus Sham group; ## *p* < 0.01 versus ICH group. Error bars, mean ± SEM. D: day.

Animals detected neuro-behavior test successively on pre-operation and post-ICH days 1, 7, and 14 to monitor neurofunction evolution. Compared to vehicle-treated mice, leptin treatment had no effect on neuro-dysfunction scores at 1d after ICH but resulted in a significant increase in scores at 7d and 14d after ICH (Figure 5a), respectively. As indicated by open field test, while leptin treatment had no effect on speeding at 1 d, it significantly increased speeding at 7 d and 14 d after ICH compared to vehicle treatment (Figure 5b). As assessed by the Grid walking test (Figure 5c) for forepaw placing and grasping, severe behavioral deficits of the left forepaw were evident in animals 1, 7, and 14 days after ICH. In contrast, animals showed significant improvement 7 and 14 days after treatment with leptin followed by ICH. Additionally, leptin-treated mice had significantly increased motor function, as assessed by rotarod test, at day 7 and day 14 after ICH, as compared to vehicle-treated mice (Figure 5d).

Thus, leptin treatment significantly promotes angiogenesis and accelerates neurofunction improvement at chronic stage of ICH.

### 3.3. Leptin Promotes Vascular Endothelial Cells Proliferation at Chronic Stage of ICH

**Figure 6:**
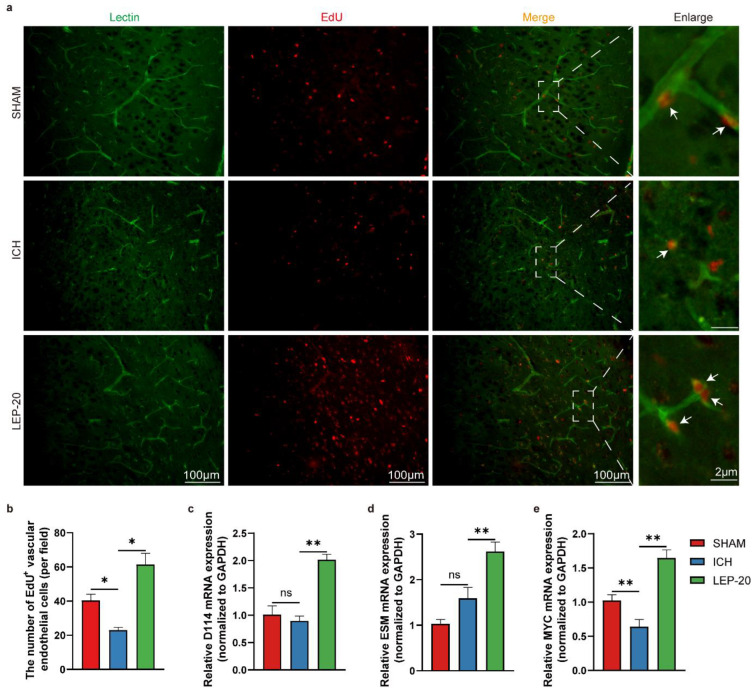
Leptin treatment promotes vascular endothelial cells proliferation at a later stage of ICH. (**a**) Representative immunofluorescence co-staining images of vascular endothelial cells marker lectin (green) and cell proliferation marker EdU (red) at day 7 after ICH. The white arrows indicate the colocalization of lectin and EdU. (**b**) Quantitative analysis of EdU immunoreactive vascular endothelial cells at day 7 after ICH. Quantification of relative gene level of sprouting endothelial tip cells markers Dll4 (**c**) and Esm (**d**) and vascular endothelial cells proliferation markers Myc (**e**) at day 7 after ICH. *n* = 6 per group. * *p* < 0.05, ** *p* < 0.01, between the indicated groups. Error bars, mean ± SEM. ns stands for non-significant.

Further, the proliferation of vascular endothelial cells was assessed. As illustrated (Figure 6a,b), vascular endothelial cells proliferated at a faster rate in leptin treatment brains than ICH mice brain cells based on EdU incorporation assay 7 days after ICH. Of note, there were no statistically significant changes between the sham group and ICH group. At the molecular level, qPCR gene expression analysis of the proliferation of vascular endothelial cells from peripheral area of hemorrhage lesion revealed a significant downregulation of Dll4 (Figure 6c) and Esm, known markers of sprouting endothelial tip cells (Figure 6d), and a strong upregulation of Myc, a powerful driver of vascular endothelial cell proliferation (Figure 6e) [40].

### 3.4. Leptin Ameliorated Pericytes Loss Induced by ICH 

**Figure 7:**
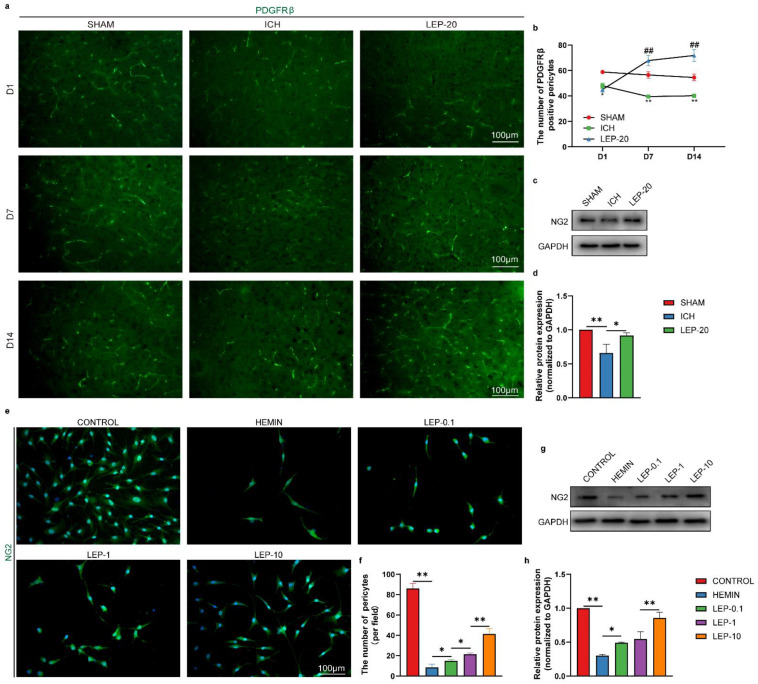
Leptin treatment promotes pericytes survival after ICH in vitro and vivo. (**a**) Representative immunofluorescence images of pericytes marker PDGFRβ at 1, 7, and 14 days after ICH. (**b**) Quantification of immunofluorescence analyses PDGFRβ at 1, 7, and 14 days after ICH. (**c**) Representative western blot figures and (**d**) quantification of relative protein level of pericytes marker NG2 at day 7 after ICH. (**e**) Representative immunofluorescence images and (**f**) quantification analyses of NG2 in HBVPC. (**g**) Representative western blot figures and (**h**) quantification of relative protein level of NG2 in HBVPC. * *p* < 0.05, ** *p* < 0.01, between the indicated groups. Especially for b, * *p* < 0.05, ** *p* < 0.01 versus Sham group; ## *p* < 0.01 versus ICH group. *n* = 6 per group. Error bars, mean ± SEM. D: day.

Previous evidence has shown that pericytes are remarkably important for the maturation and stabilization of blood vessels during angiogenesis [42]. Then, antibodies specific for pericytes (PDGFRβ and NG2, green) were used to assess the pericytes survival in the peripheral area of hemorrhage lesion. As shown in Figure 7a,b, ICH mouse exhibited significantly decreased expression of the pericyte marker PDGFRβ. Additionally, western blot revealed markedly reduced expression of another pericyte marker, NG2, in the ICH group. Nevertheless, these changes were ameliorated in the leptin-treated mouse (Figure 7c,d). To further verify the above observations, we subjected human brain microvascular pericytes (HBVPC) to the hemorrhage-like insult. After hemin exposure, both with immunofluorescence and western blot, the expression of NG2 in HBVPC was significantly decreased. As expected, leptin also reversed hemin-induced pericyte loss in HBVPC (Figure 7e–h).

### 3.5. Leptin Enhances Pericytes Proliferation and Migration after ICH

**Figure 8:**
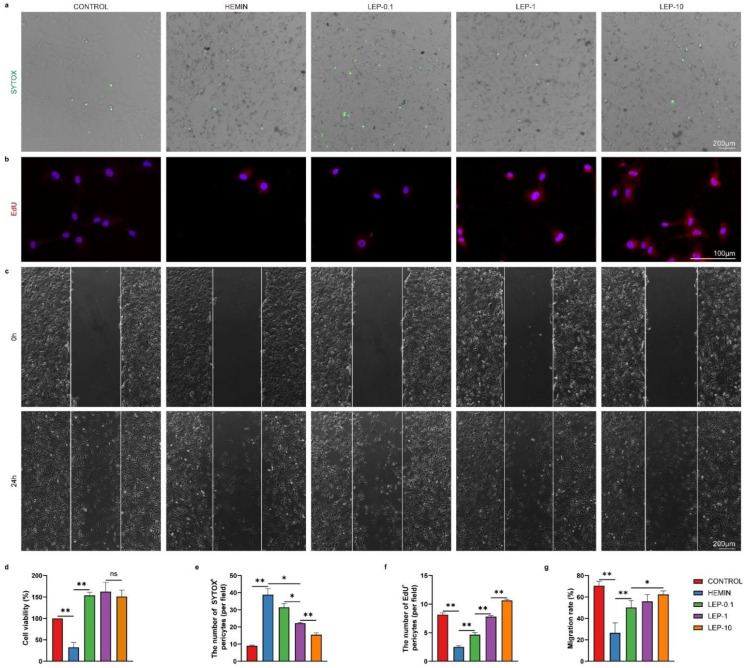
Leptin treatment promotes pericytes’ survival, proliferation, and migration after ICH in vitro. (**a**) Representative images and (**e**) quantification analyses of SYTOX staining in HBVPC. (**b**) Representative images and (**f**) quantification analyses of EdU staining in HBVPC. (**c**) Representative images and (**g**) quantification analyses of HBVPC migration at o hours and 24 h after wound scratching. (**d**) Cell survival was analyzed after injury with or without leptin treatment by CCK-8 assay. *n* = 6 per group. * *p* < 0.05, ** *p* < 0.01 between the indicated groups. Error bars, mean ± SEM. ns stands for non-significant.

Next, further study was undertaken to access the effect of the leptin on pericyte survival. To further explore the effect of leptin treatment in hemin induced HBVPC injury, cell viability was quantified with CCK-8 assay and SYTOX Green nucleic acid stain. As shown in Figure 8a,d,e, cell viability significantly decreased in HBVPC under hemorrhage condition. In contrast, leptin enhanced pericyte viability in a dose-dependent manner. We next examined the proliferation of HBVPC using EdU incorporation. We observed that the percentage of EdU-positive cells in HBVPC with hemin treatment was significantly lower than that in normal condition. In contrast, the percentage of EdU-positive HBVPC was significantly enhanced in the presence of leptin exposure compared with that of hemin treatment cells. Thus, leptin positively influenced the proliferation of HBVPC (Figure 8b,f). Migration of pericyte is fundamental to cover and stabilize newly formed blood vessels. Next, we assessed the migration of HBVPC using scratch assay. Leptin treatment led to accelerated migration of pericytes when compared to hemin-treated HBVPC. In contrast, hemin-treated HBVPC did not respond to the promigratory effects when compared to normal condition (Figure 8c,g).

### 3.6. Pericytes-Derived Angiogenic Factors Are Responsible for Leptin Proangiogenic after ICH

**Figure 9:**
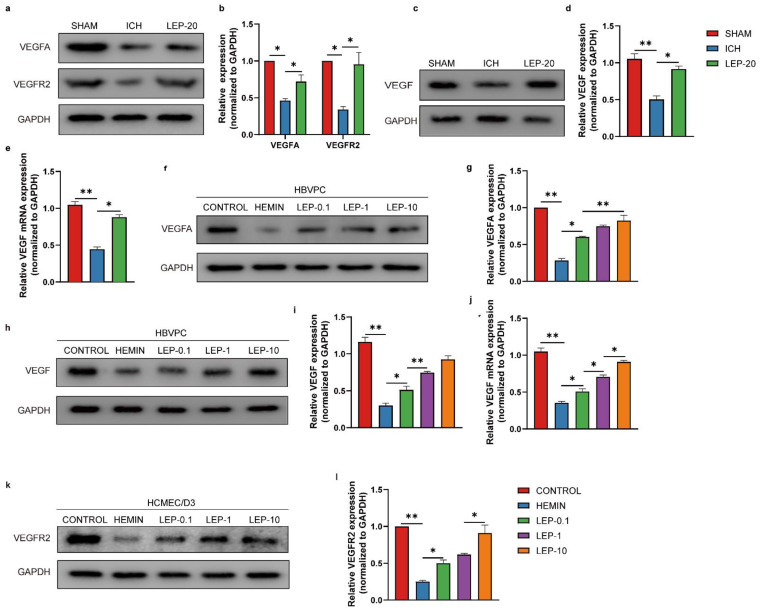
Pericytes-derived pro-angiogenic chemokines are responsible for vascular regeneration following leptin treatment with ICH. (**a**) Representative western blot figures and (**b**) quantification of relative proteins level of pro-angiogenic chemokines VEGFA and VEGFR2 at day 7 after ICH. (**c**) Representative western blot figures and (**d**) quantification of relative protein level of pro-angiogenic chemokines VEGF at day 7 after ICH. (**e**) Quantification of relative mRNA level of VEGF at day 7 after ICH. (**f**) Representative western blot figures and (**g**) quantification of relative protein level of pro-angiogenic chemokines VEGFA2 in HBVPC. (**h**) Representative western blot figures and (**i**) quantification of relative protein level of VEGF in HBVPC cells in indicated groups. (**j**) Quantification of relative mRNA level of VEGF in HBVPC cells in indicated groups. (**k**) Representative western blot figures and (**l**) quantification of relative protein level of VEGFR2 in HCMEC/D3 cells in indicated groups. *n* = 3 per group. * *p* < 0.05, ** *p* < 0.01, between the indicated groups. Error bars, mean ± SEM.

Extensive literature has shown that pericytes produce various trophic factors, such as vascular endothelial growth factor VEGFA, that are involved in angiogenesis. Additionally, VEGFA was reported to promote angiogenesis via upregulating VEGFR2 in ECs, an isoform receptor for VEGFA [43]. Therefore, the expression levels of total VEGF, VEGFA, and VEGFR2 proteins were assessed in ICH model both in vivo and in vitro. As result shown in Figure 9a–d, the expression of the total VEGF, VEGFA, and VEGFR2 were markedly reduced following the brain ICH induction compared with sham, which could be significantly reversed by leptin treatment. In agreement with our observations in vivo, both total VEGF and VEGFA in pericytes (Figure 9f–i) and VEGFR2 in ECs (Figure 9k,l) expression were significantly decreased at 24 h following hemin treatment. As expected, leptin treatment markedly increased hemin-induced VEGFA and VEGFR2 expression. This tendency was also found in the total VEGF mRNA level in ICH model both in vivo and in vitro (Figure 9e,j).

**Figure 10:**
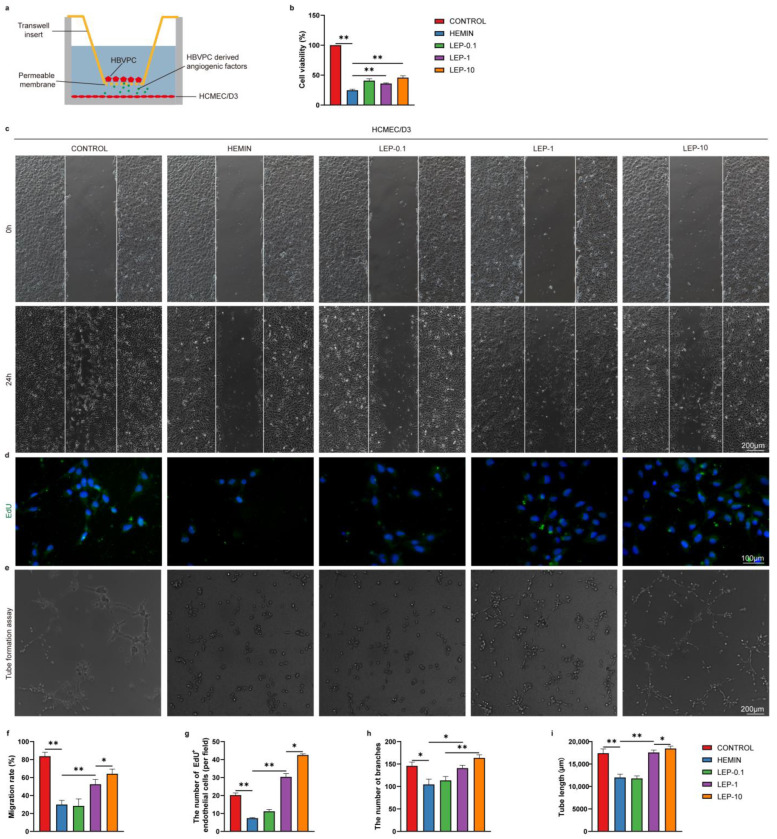
Pericytes-derived pro-angiogenic factors enhance the survival, migration, proliferation, and tube formation of vascular regeneration following leptin treatment in vitro. (**a**) Schematic representation of Pericyte and endothelial cell co-culture system. (**b**) Cell survival was analyzed by CCK-8 assay. (**c**) Representative images and (**f**) quantification analyses of HCMEC/D3 migration at 0 h and 24 h after wound scratching. (**d**) Representative images and (**g**) quantification analyses of EdU staining in HCMEC/D3. (**e**) Images of the endothelial tubular networks in Matrigel after 24 h. Analyses of tube formation were done with image J software using the Angiogenesis Analyzer Plugin. Quantification analyses data were shown in (**h**, **i**). *n* = 3 per group. * *p* < 0.05, ** *p* < 0.01, between the indicated groups. Error bars, mean ± SEM.

We next set out to determine whether pericyte-secreted these factors may regulate the ECs survival, migration, proliferation, and tube formation by cocultured with pericytes versus ECs without direct contact by the use of a Transwell system (Figure 10a). This system allows us to monitor the HCMEC/D3 cell viability, migration, proliferation, and tube formation mediated by pericyte-secreted angiogenic factors. Firstly, the cell viability in HCMEC/D3 was determined by CCK-8 assay. As shown in Figure 10b, cell viability was decreased in hemin-injured HCMEC/D3 cells. As expected, leptin treatment significantly increased HCMEC/D3 cell viability to a larger extent than that with hemin alone did. Next, the proliferation in HCMEC/D3 was determined by EdU assay. As shown in Figure 10d,g, hemin-induced cell injury decreased the EdU-positive cells, which could be significantly reversed following leptin treatment. A scratch healing assay was used to investigate HCMEC/D3 migration. As shown in Figure 10c,f, HCMEC/D3 migration capacity was reduced after hemin treatment. Similarly, leptin exposure markedly increased the migration of HCMEC/D3. Furthermore, the formation of tube-like structures was observed. Interestingly, the formation of endothelial tube-like structures was significantly accelerated in the presence of leptin compared with hemin alone (Figure 10e,h,i).

### 3.7. Pericyte Leptinr/STAT3 Signaling Pathway Is Responsible for Leptin Angiogenic after ICH

**Figure 11:**
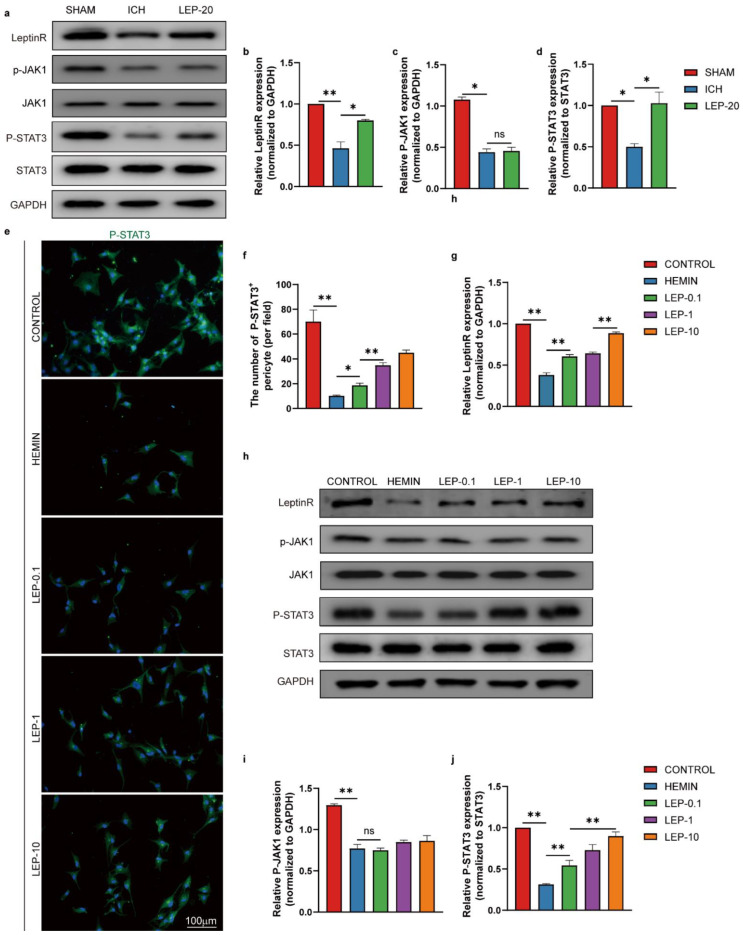
Pericyte leptinR/STAT3 signaling pathway is responsible for leptin angiogenic actions in ICH. (**a**) Representative western blot figures and (**b**–**d**) quantification of relative protein level of leptinR, p-JAK1,P-STAT3 at day 7 after ICH. (**e**) Representative immunofluorescence images and (**f**) quantification of immunofluorescence analyses of P-STAT3 in HBVPC. (**h**) Representative western blot figures and (**g**,**i**,**j**) quantification of relative protein level of leptinR, p-JAK1,P-STAT3 in HBVPC. *n* = 3 per group. * *p* < 0.05, ** *p* < 0.01, between the indicated groups. Error bars, mean ± SEM. ns stands for non-significant.

We next reasoned that leptin downstream signaling pathways may play a vital role in pericyte beneficial effects on angiogenesis after ICH. Extensive literature has shown that leptin binding induces activation of Janus Kinase (JAK) and signal transducers and activators of transcription (STATs), particularly STAT3 [44]. In addition, STAT3 is a critical transcription activator in angiogenesis [45]. Thus, we assessed leptinR/STAT3 signaling pathway during ICH. Both total leptinR and phosphorylation of STAT3 protein level were shown significantly decreased in ICH mice. However, leptin treatment significantly increased these protein expression as compared to ICH mice. In contrast, no significant difference in protein levels of phosphorylated JAK was identified after treatment with leptin in ICH mice. (Figure 11a–d). As shown in Figure 11e–j, upon hemin stimulus, total leptinR and phosphorylation of STAT3 protein level significantly reduced in HBVPC. Importantly, incubation with leptin notably increased protein expression of leptinR and phosphorylation of STAT3 after hemin-induced HBVPC injury in a dose-dependent manner. However, leptin have not altered phosphorylated JAK level in hemin-induced HBVPC injury. These results suggest that leptin may result in angiogenesis after ICH via activation pericyte leptinR/STAT3 signaling pathway.

### 3.8. STAT3 Inhibition Reverses the Leptin Pro-Angigenic Effect after ICH

**Figure 12:**
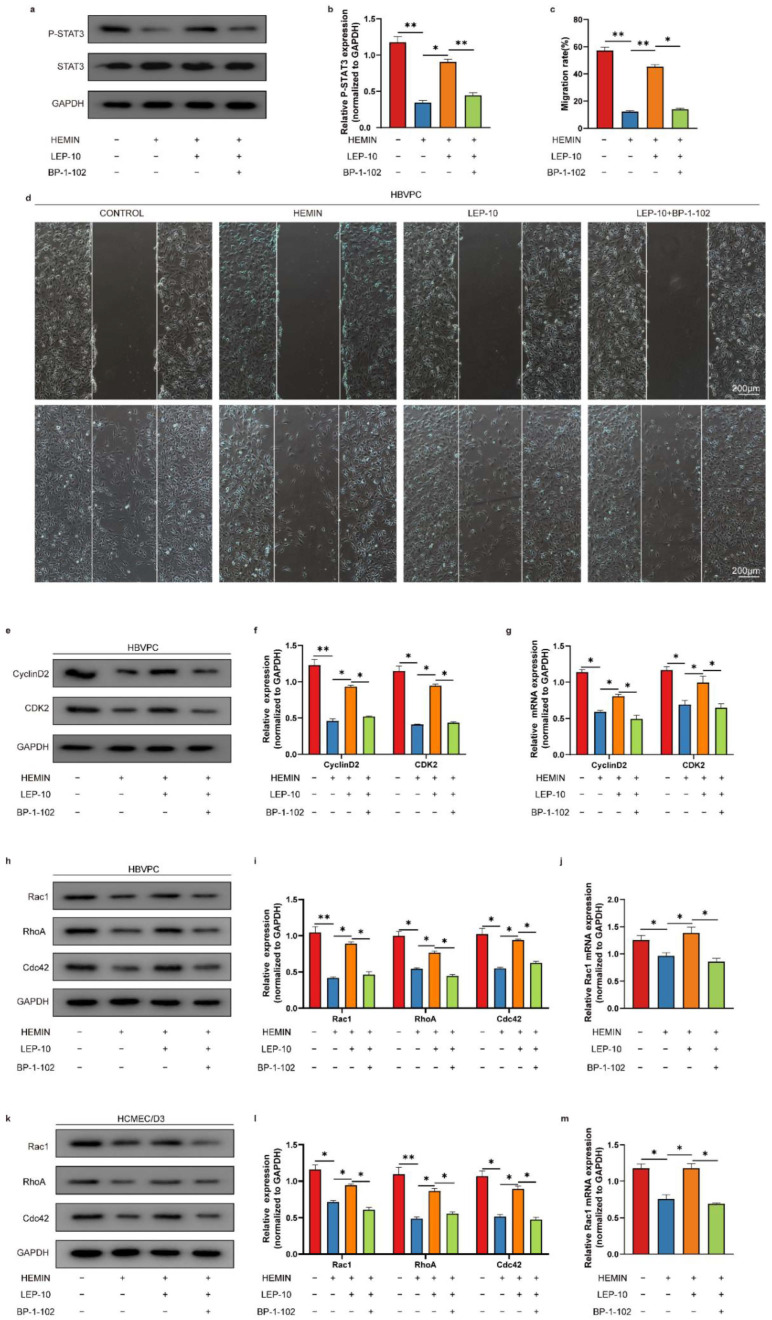
STAT3 inhibition reverses the pro-angiogenic effect of leptin following ICH. (**a**) Representative western blot figures and (**b**) quantification of relative protein level of P-STAT3 in HBVPC cells in indicated groups. (**c**) Representative images and (**d**) quantification analyses of in HBVPC cells migration at 24 h after wound scratching. (**e**,**h**) Representative western blot figures and and (**f**) quantification of relative protein level of CyclinD2 and CDK2 in HBVPC cells in indicated groups, and (**i**) quantification of relative protein level of Rac1, RhoA, and Cdc42 in HBVPC cells in indicated groups. (**g**,**j**) Quantification of relative mRNA level of CyclinD2, CDK2, and Rac1 in HBVPC cells in indicated groups. (**k**) Representative western blot figures and (**l**) quantification of relative protein level of Rac1, RhoA, and Cdc42 in HCMEC/D3 cells in indicated groups. (**m**) quantification of relative mRNA level of Rac1 in HCMEC/D3 cells in indicated groups. *n* = 3 per group. * *p* < 0.05, ** *p* < 0.01, between the indicated groups. Error bars, mean ± SEM.

We next tested whether STAT3 directly modulated leptin pro-angiogenic effect following ICH. STAT3 inhibition with a specific antagonist, BP-1-102, resulted in a significantly decreased STAT3 protein levels in pericytes after ICH (Figure 12a,b). We hence interrogated the effect of STAT3 inhibition on pericytes and ECs proliferation and migration. In wound healing assay, pericytes migration rate was remarkably increased in leptin treated cells as compared with model cells. However, it was significantly decreased after combination BP-1-102 with leptin treatment in pericytes (Figure 12c,d). As seen in Figure 12 e–m, the representative proliferation (CyclinD2, and CDK2) and migration (Rac1, RhoA and Cdc42)-related molecules were also detected using western blot and qPCR. The protein levels of CyclinD2, CDK2, Rac1, RhoA, and Cdc42 were significantly lower in hemin-induced pericytes injury model group than that in the control group. Meanwhile, treatment with leptin demonstrated the ability to promote those proteins compared with that of the model group. However, combination BP-1-102 with leptin treatment markedly reversed it (Figure 12e,f,h,i). Additionally, in comparison to the control group, the hemin-induced pericytes injury model group had significantly reduced mRNA levels of CyclinD2, CDK2, and Rac1. When compared to the model group, leptin therapy showed an improved ability to stimulate those genes. However, treatment with BP-1-102 and leptin together significantly restored it (Figure 12g,j). Furthermore, hemin-induced ECs damage model group Rac1, RhoA, and Cdc42 protein levels were considerably lower than those in the control group. In contrast to the model group, leptin administration showed the potential to promoted higher levels of those proteins. In contrast, treatment with BP-1-102 and leptin together great reversed it (Figure 12k,l). The mRNA level of Rac1 also markedly decreased after combination BP-1-102 with leptin treatment in ECs (Figure 12m).

### 3.9. Leptin Alleviates Hostile Microenvironment at Chronic Stage of ICH

**Figure 13:**
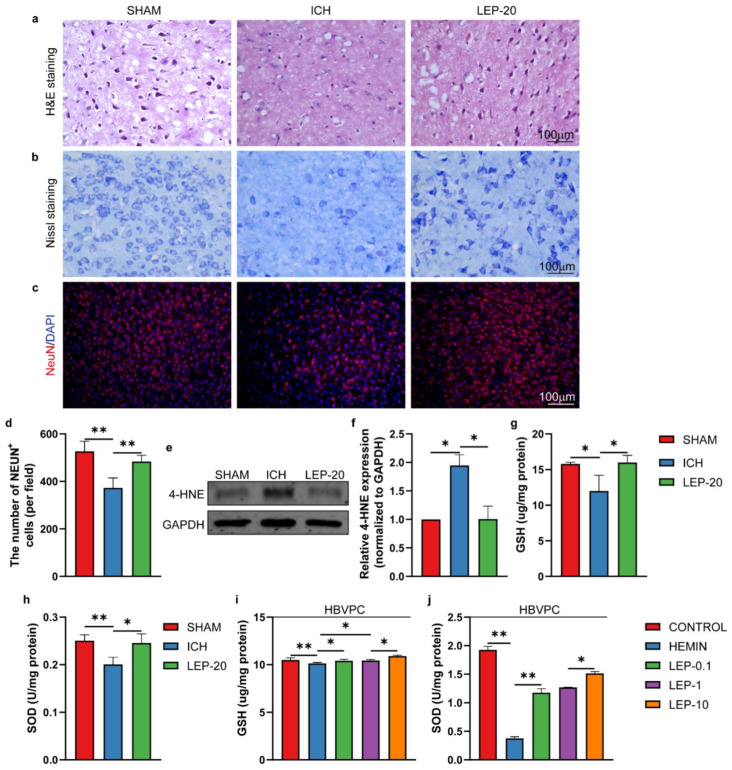
Leptin treatment alleviates hostile microenvironment at a later stage of ICH. (**a**) Representative HE staining sections of the hemorrhage region at day 7 after ICH. (**b**) Representative Nissl staining in the hemorrhage region at day 7 after ICH. (**c**) Representative immunofluorescence co-staining images and (**d**) quantification of immunofluorescence analyses of neuron marker NeuN (red) and nucleus marker DAPI (blue) in the hemorrhage region at day 7 after ICH. (**e**) Representative western blot figures and (**f**) quantification of relative protein level of 4-HNE at day 7 after ICH. (**g**,**h**) Quantification of the GSH and SOD level at day 7 after ICH. (**i**,**j**) Quantification of the GSH and SOD level in HBVPC. *n* = 3 per group. * *p* < 0.05, ** *p* < 0.01, between the indicated groups. Error bars, mean ± SEM.

Following ICH, microenvironment mitigation has vital results for angiogenesis. Therefore, we assessed the pathological changes in the perihematomal region after ICH. As the results show, the number of Nissl-positive cells was lower, and the tissue damage was higher in perihematomal brain tissue of ICH mice than that in sham mice. In contrast, leptin treatment dramatically attenuated tissue damage and increased Nissl-positive cells 7 days after ICH, as indicated by H&E staining (Figure 13a) and Nissl’ s staining (Figure 13b), respectively. In addition, we also observed the survival neurons in perihematomal region after ICH. As the results show, the number of neurons markedly decreased in ICH mice compared to sham mice. However, it was significantly reversed with leptin treatment (Figure 13c,d). Furthermore, we assessed the status of lipid peroxidation by detecting the level of 4-HNE. As the results in Figure 13e,f show, ICH mice exhibited a significantly elevated level of 4-HNE adducts proteins compared with sham mice. Leptin treatment decreased 4-HNE content to the level in sham group. Next, the influence of leptin on oxidative stress under hemorrhage condition was also studied. As a result, hemorrhage results in decreasing of the anti-oxidative stress marker GSH (Figure 13g,i) and SOD (Figure 13h,j) in vivo and vitro. However, treatment with various quantities of leptin markedly reversed these generations.

## 4. Discussion

ICH is the most destructive type of stroke, leading to severe cerebral hematoma, peripheral tissue edema, and secondary nerve cell injury tissue defects [46]. Angiogenesis is an important compensatory mechanism to protect brain nerve function after ICH [47]. The establishment of new vessels holds promise for vascular perfusion, energy supply, and brain self-repair. Previous studies also observed numerous newborn microvessels around the hematoma [48]. In the present study, we showed that exogenous leptin markedly promoted ECs proliferation and survival in a dose-dependent manner in vivo and in vitro after ICH (Figure 2, Figure 4, Figure 6 and Figure 10). Moreover, the enhanced angiogenesis after leptin treatment contributes to this neuroprotection, since a remarkable reduction of neurological dysfunction and hostile microenvironment is showed at a late phase of ICH (Figure 3, Figure 5 and Figure 13). These results thus indicate that leptin is an important angiogenic mediator in the brain following ICH. However, extensive studies focusing on effects of leptin on ICH have shown paradoxical evidence. On one hand, clinical observations have indicated that high serum leptin levels were associated with a poor functional recovery in ICH patients [49]. In addition, leptin was also found to be a novel mediator of the inflammation and played a critical role in detriment secondary brain injury after ICH mice [50]. On the other hand, a recent Framingham study’s results showed that leptin levels were not directly related to the risk of all stroke incidents, including ICH [51]. Moreover, plasma leptin levels in ICH patients increased during the 6 h immediately, peaked in 24 h, and decreased gradually thereafter [52]. Noteworthily, it has been shown that exogenous administration of leptin only worsens early phase of secondary brain injury after ICH mice [50]. A possible angiogenesis effect of leptin at chronic phase of ICH was not evaluated in these studies. Contrarily, our findings show that leptin has a sizable pro-angiogenic effect at the late stages of ICH. We suggest some explanations for these surprising findings: (1) A distinct method of drug administration. While intraperitoneal injection was frequently employed in earlier studies, we have adopted lateral ventricle injection to prevent the unneeded effects of leptin on areas beyond the central nervous system. (2) The amount of leptin used in this trial was significantly decreased because we used the intracerebro-ventricular injection technique. (3) Since leptin’s effects on ICH may vary depending on when they occur, we concentrated on how it affected the latter stages of the disease. (4) Leptin may induce a significant increase in the levels of certain inflammatory factors in the early stage of ICH, while it may induce the increase of some endogenous repair factors in the late ICH. Additionally, this effect may be closely dependent on the brain leptin level. The related mechanism needs to be further explored. Regardless, given the limited endogenous angiogenesis after ICH, our study provides new insights into leptin as a viable candidate target for neurological recovery. 

Since leptinR/Jak-STAT signaling pathway has been found to be related to the angiogenesis through leptin in stroke [53] and tumors [54], we are interested in whether leptin promotes angiogenesis in the context of ICH through the activation of this pathway. Indeed, our results also demonstrate that an exogenous leptin infusion significantly increased leptinR as well as phosphorylated STAT3 level both in vivo and in vitro after ICH; however, phosphorylated Jak level did not alter significantly either in vivo or in vitro after ICH (Figure 11 and Figure 12). Additionally, VEGFA and VEGFR2, one of the most important proangiogenic growth factors, were markedly increased after leptin treatment both in vivo and in vitro ICH model (Figure 9). Therefore, we speculate that leptin signaling activation leads to phosphorylation of leptin receptor. Phosphorylate of leptin receptor serves as a binding site for STAT3 protein. Leptin signaling also results in STAT3 binding. After STAT3 recruitment to leptinR, STAT3 becomes tyrosine-phosphorylated by JAK2, which leads to dissociation from the receptor and dimerization. STAT3 dimers then translocate into the nucleus and act as transcription factors by binding to specific response elements in the promoter of their target genes, such as VEGF. Taken together, our results suggest that, following ICH, leptin induced angiogenesis following ICH, typically through activation of leptinR/STAT pathway and subsequent proangiogenic growth factors release. 

It is worth noting that leptin treatment simultaneously attenuated ICH-induced neuro-dysfunction at a later stage. However, according to previous studies about the role of leptin in the early phase of secondary brain injury after ICH, leptin has a detrimental effect on ICH and is partly involved in promoting inflammatory process [55,56]. The protective or harmful effect of leptin after various diseases is not seen uniformly, and we speculate that it depends on the stages of ICH, doses of leptin, and action timing. Moreover, inhibition of leptin/STAT3 signaling pathway could not reduce secondary brain injury after ICH. This result further indicates that leptin has other roles in contributing to ICH besides proinflammatory effect.

Numerous reports describe an important role for pericyte in angiogenesis [57]. However, the specific role of leptin in pericyte under ICH condition was previously unknown. A recent finding showed that pericytes expressed the leptin receptor [37]. In the present study, we found that exogenous leptin treatment promoted pericytes survival, proliferation, and migration, and pro-angiogenic chemokines release in vitro and in vivo model of ICH (Figure 7, Figure 8 and Figure 9). Furthermore, leptin treatment enhanced the ECs survival, proliferation, sprouting, and tube formation, dependent on pericytes under ICH condition (Figure 6 and Figure 10). Together, our data suggest that pericytes play a vital role in neuroprotection of leptin treatment after ICH. However, the detail regulation mechanisms are still elusive.

**Figure 14:**
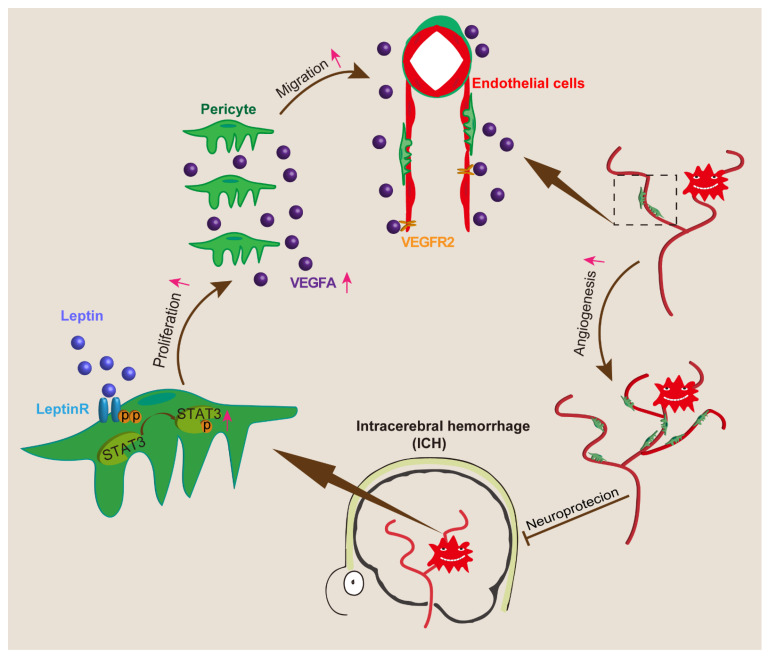
Schematic summary of the effect of underlying leptin, which drives remarkable angiogenesis and alleviates neurological dysfunction post ICH.

In conclusion, the major findings of the current study are as follows: (1) an exogenous leptin injection promoted neurofunction recovery after ICH, (2) leptin promoted ICH-induced angiogenesis, and (3) leptin-induced angiogenesis after ICH via pericyte leptinR/STAT3 pathway. A proposed angiogenesis and signaling pathway for leptin action according to our study is shown in Figure 14. We believe that the perihematomal angiogenesis mediated by leptin can be a new therapeutic target for ICH treatment.

## Data Availability

The raw data supporting the conclusions of this manuscript will be made available by the authors, without undue reservation, to any qualified researcher.
